# The History of Capabilities of Herbal Simples

**Published:** 1891-07-18

**Authors:** W. T. Fernie


### The History and Capabilities of Herbal Simples.

By W. T. Fernie, M.D.

### XXXV.—EYEBRIGHT.

Found in abundance in summertime on our heaths, and on mountains near the sea this elegant little plant serves to explain a paradox of nursery tradition. "There was a man of Thessaly," says the well-remembered rhyme, "and he was wondrous wise; He jumped into a quickset hedge, And scratched out both his eyes. But when he saw his eyes were out, With all his might and main He jumped into another hedge, and scratched them in again." Growing freely in, or about that second hedge must have been the timely eyebright —Euphrasia Officinalis—famous from earliest times for preserving and restoring the sight. The Greeks named this plant originally from the linnet which first made use of the herb for clearing its vision, and which, subsequently, gave the same notion to mankind. Also the Greek word which gives its title to the eyebright signifies joy and gladness. Arnoldus, in his *Lilium Medicinæ*, 1305, recorded its medicinal properties as cephalic, ophthalmic, and good for a weak memory. Shenstone declared "Famed Euphrasy may not be left unsung, Which grants dim eyes to wander leagues around." And Milton has told us, in *Paradise Lost*, that

> "Michael from Adam's eyes the film removed;
> Then straightway purged with Euphrasy and Rue
> The visual nerve; for he had much to see."

"Eyebright," said Culpepper, "made into a powder, and then into an electuary with sugar, hath powerful effect to help and to restore the sight decayed through years; and if the herb were but as much used as it is neglected it would long since have spoiled the trade of the spectacle maker." Hildanus relates that this plant restored the sight of many persons at the age of seventy, or eighty years. And, on the whole, it seems most probable that the eyebright has been found to succeed best for eyes weakened by long-continued straining, and for those which are dim and watery from old age. Nevertheless, some sceptical modern writers do not hesitate to say that the eyebright owes its reputation solely to the fact that its tiny flower bears in its centre a yellow spot darker towards the middle, and closely resembling the human eye; so that from this circumstance, on the doctrine of signatures, it was pronounced curative of all visual derangements. For a like reason the natural order to which the plant belongs to botanically has been named Scrophularineous, or scurvy-curing. But on positive experimental evidence one of our dominant medical schools assures us to-day the eyebright has undoubtedly a distinct, though limited sphere of curative action within which it manifests virtues which are as unvarying as they are truly potential. It acts specifically on the mucous lining of the eyes and nose and uppermost throat to the top of the wind-pipe, causing, when given in excess, a profuse secretion from these parts; whilst curing the same troubles when arising from catarrh if given in smaller doses. A medicinal tincture is made from the whole plant, and can be readily obtained from our leading druggists. In simple inflammation of the eye, with a blood-shot condition of its outer coats, this tincture, if used with rosewater as a lotion, and given at the same time as a medicine, will almost certainly cure the ailment. Thirty drops should be added to a wineglassful of rosewater for making the lotion; and three or four drops of the tincture should be given with a tablespoonful of cold water three times in the day as a medicine. What precise chemical constituents occur in the eyebright beyond tannin, mannit, and glucose are not yet recorded. In Iceland its expressed juice is used for most diseases of the eyes. Likewise in Scotland the Highlanders infuse the plant in milk, and employ this for bathing weak or inflamed eyes. In France the herb is called *casse lunettes*; and in Germany *Augen trost.* As an incidental matter of interest respecting weak eyes I may state that among the Gipsy race a cure for ophthalmia is wrought by the grandmother "producing dried earth from her medicine box, and after spitting on this she works it up with her finger tip into a thin paste, with which the child's eyes are anointed, and then bound round with a piece of rag kept wet for three days with water in which a cabbage has been boiled." In confirmation of which remedy we reverently read in the Gospel of St. John about a man "which was blind from his birth," and with regard to whom our Saviour "spat on the ground, and made clay of the spittle, and anointed the eyes of the blind man with the clay." More than one modern oculist of eminence has advised that weak eyes should be wetted daily with the fasting saliva, for strengthening and benefiting these organs. In old days the saliva lustralis was applied to the forehead of infants with the third finger to protect them against the evil eye. Nowadays in Scotland the same end is sought to be attained by twisting a red silk thread around the forefinger, or by wearing a slip of mountain ash in the bonnet, or by eating Skaith Saw, a gruel, "thick, and slab, and detestable to witches." The Arabians knew the plant eyebright under the name Adhil. With ourselves it forms an ingredient of British herbal tobacco, which is admirably useful for chronic colds in the head and upper chest.

